# Induced osteogenic differentiation of human smooth muscle cells as a model of vascular calcification

**DOI:** 10.1038/s41598-020-62568-w

**Published:** 2020-04-06

**Authors:** Wera Pustlauk, Timm H. Westhoff, Luc Claeys, Toralf Roch, Sven Geißler, Nina Babel

**Affiliations:** 1 Berlin Institute of Health Center for Regenerative Therapies (BCRT), Charité - Universitätsmedizin Berlin, corporate member of Freie Universität Berlin, Humboldt-Universität zu Berlin, and Berlin Institute of Health, Berlin, Germany; 2 Julius Wolff Institute, Charité - Universitätsmedizin Berlin, corporate member of Freie Universität Berlin, Humboldt-Universität zu Berlin, and Berlin Institute of Health, Berlin-Brandenburg Center for Regenerative Therapies, Berlin, Germany; 3 Berlin-Brandenburg School for Regenerative Therapies, Charité - Universitätsmedizin Berlin, corporate member of Freie Universität Berlin, Humboldt-Universität zu Berlin, and Berlin Institute of Health, Berlin, Germany; 4 Center for Translational Medicine, Department of Internal Medicine I, Marien Hospital Herne, University Hospital of the Ruhr-University Bochum, Bochum, Germany; 5 Department of Vascular Surgery, Marien Hospital Herne, University Hospital of the Ruhr-University Bochum, Bochum, Germany

**Keywords:** Cell biology, Mechanisms of disease, Medical research, Experimental models of disease, Cardiovascular biology, Cardiovascular diseases

## Abstract

Vascular calcification is a severe pathological event in the manifestation of atherosclerosis. Pathogenic triggers mediating osteogenic differentiation of arterial smooth muscle cells (SMC) in humans remain insufficiently understood and are to a large extent investigated in animal models or cells derived thereof. Here, we describe an *in vitro* model based on SMC derived from healthy and diseased humans that allows to comprehensively investigate vascular calcification mechanisms. Comparing the impact of the commonly used SMC culture media VascuLife, DMEM, and M199, cells were characterised by immunofluorescence, flow cytometry, qPCR, and regarding their contractility and proliferative capacity. Irrespective of the arterial origin, the clinical background and the expansion medium used, all cells expressed typical molecular SMC marker while contractility varied between donors. Interestingly, the ability to induce an osteogenic differentiation strongly depended on the culture medium, with only SMC cultured in DMEM depositing calcified matrix upon osteogenic stimulation, which correlated with increased alkaline phosphatase activity, increased inorganic phosphate level and upregulation of osteogenic gene markers. Our optimized model is suitable for donor-oriented as well as broader screening of potential pathogenic mediators triggering vascular calcification. Translational studies aiming to identify and to evaluate therapeutic targets in a personalized fashion would be feasible.

## Introduction

Vascular calcification due to osteogenic differentiation of arterial smooth muscle cells (SMC) is a severe pathological event in atherosclerosis. Massive, bone-like or brittle, amorphous calcifications can occlude the lumen, cause atherosclerotic plaque rupture, thrombus formation and infarction or result in failure of the arterial wall^1–6^. Deposition of calcified matrix within the vessel wall was described as an active, SMC-driven process^5,7^. This process is initially marked by the switch of SMC from their contractile phenotype towards a synthetic state, in which proliferation and migration is enhanced^8–12^. The ability of SMC for phenotypic switching^1,9^ is important during vascular repair, but makes them also susceptible to pathological changes^8,9,12–14^, including their differentiation into osteoblast- and chondrocyte-like cells or acquisition of macrophage and foam cell markers^1,11,13–22^.

Several studies investigated the processes and key factors involved in the osteogenic differentiation of mammalian SMC, subsequent deposition of mineralized matrix and vascular calcification^20,21,23–25^. These studies provide evidence that an active inhibition of calcification and the prevention of pro-osteogenic gene expression is crucial for retention of the SMC phenotype^7,23,26–28^. Conversely, it was found that SMC-mediated calcification is enhanced by an inflammatory environment, oxidative stress, hypercalcaemia and hyperphosphatemia, as well as apoptosis, and senescence^1,25,29–31^. Despite the fact that these pathogenic triggers link atherosclerosis with other disorders like diabetes, chronic kidney disease, and osteoporosis, the specific mechanism underlying the osteogenic differentiation of SMC are still insufficiently understood^1,7,32,33^.

Since atherosclerosis and vascular calcification remain the leading cause of death worldwide there is a huge need to shift the research focus from the prevention of the disease towards late-stage intervention strategies for patients with already established disease and calcified plaques^34^. Considering this high medical demand and the often inadequate transferability of animal data to the clinically relevant human situation, especially innovative human *in vitro* models are needed. Studies of underlying disease mechanisms in the human context using biological material from the relevant patient cohorts will, furthermore, support the translation of the findings into clinical application. Such suitable human *in vitro* models require reproducible isolation and characterization of SMC from healthy and diseased arteries to enable the investigation of the pathological mechanism underlying SMC osteogenic transdifferentiation. Although, various *in vitro* approaches were used in previous studies, a comprehensive human model that includes systematic characterisation of SMC derived from healthy and diseased human donors is missing. Furthermore, systematically optimized culture conditions to investigate the osteogenic differentiation of SMC and a conclusive comparison of commonly used SMC culture media including their impact on the SMC phenotype is lacking.

Here, we established a human *in vitro* model of SMC calcification based on primary human SMC that allows to study the impact of potential mediators such as patient sera, immune cells, cytokines, drugs, or inhibitors on vascular calcification. We developed a protocol for the isolation of SMC derived from clinical samples of patients with various pathological alterations. The obtained SMC were analysed for their marker expression, contractility, proliferation, and osteogenic differentiation potential under different cell culture conditions. Our in depth evaluation of SMC derived from six human donors demonstrates that this *in vitro* model with its comprehensive SMC characterisation and detailed analysis of the osteogenic differentiation process is suitable not only for the broad screening of potential pathogenic mediators triggering vascular calcification, but also for the identification of new therapeutic targets in a personalized fashion.

## Results

### Isolated cells display SMC characteristics irrespective of their arterial origin and clinical background

**Figure 1:**
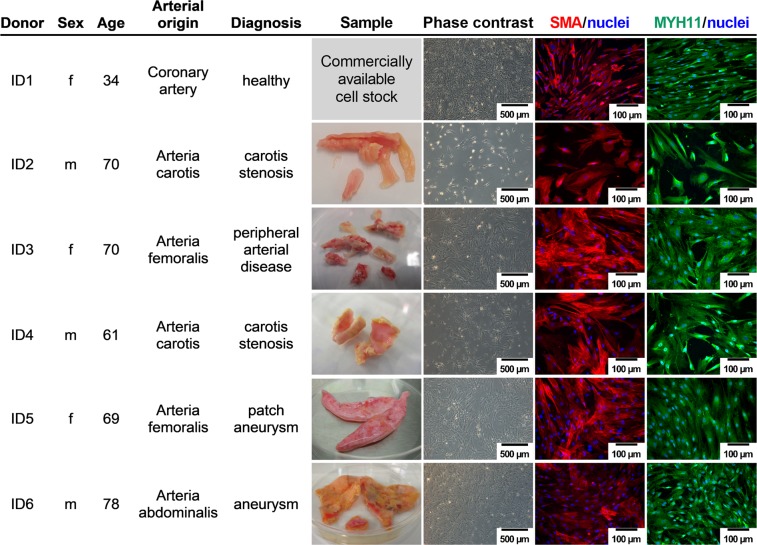
Donor characteristics, sample overview, and immunofluorescence characterisation of isolated SMC. Cells isolated from various pathologically altered, clinical samples of different arterial origin were analysed morphologically and by immunofluorescence staining of specific marker proteins to confirm their SMC characteristics at passage three. Nuclei (blue) were visualized with DAPI. SMA (red) - smooth muscle actin, and MYH11 (green) - myosin heavy chain 11 were labelled with the respective antibodies.

Primary cells isolated from various pathologically altered, clinical samples of different arterial origin were analysed with regard to their SMC phenotype as well as their SMC characteristics and compared to corresponding commercially available SMC isolated from healthy coronary arteries. Phase contrast images acquired at passage three showed a typical SMC morphology (Fig. 1). Cell identity was further confirmed by positive immunofluorescence staining for smooth muscle actin (SMA) and myosin heavy chain 11 (MYH11). No differences in SMA and MYH11 expression between healthy coronary artery SMC and cells derived from pathologically altered samples were observed. In addition, all SMC isolations were negative for the human skeletal muscle myoblast marker myosin heavy chain 4 (Supplementary Fig. S1). Thus, successful SMC isolation even from heavily calcified arteries demonstrates suitability of the combined approach of tissue digestion and explant culture, irrespective of the clinical background, the arterial location, gender or patient age.

### Isolated cells show SMC characteristics irrespective of the expansion medium used

**Figure 2:**
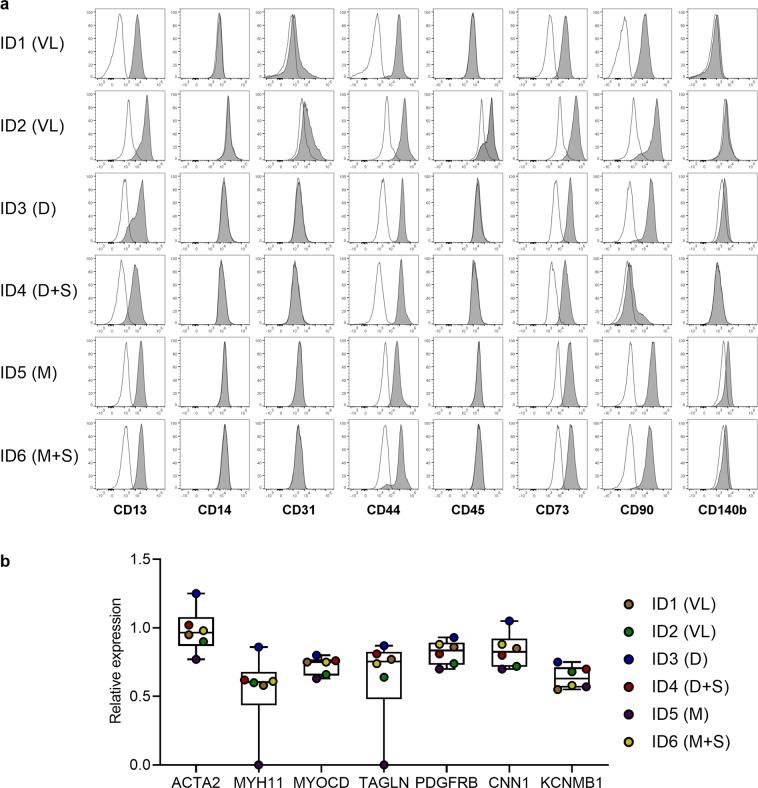
SMC marker expression after expansion in different media. (**a**) Histograms of flow cytometric surface marker expression (grey) of CD13, CD14, CD31, CD44, CD45, CD73, CD90, and CD140b on single live cells at passage three or four identified according to the gating strategy shown in Supplementary Fig. S2. Marker expression is given in comparison to the unstained control (white). (**b**) Gene expression level of SMC marker in isolated cells at passage one or two, normalized to the expression of the housekeeping gene *RPL13A*. Values of the individual donors are shown as points superimposed onto the boxplots. Expansion medium of each donor is given in brackets; D - DMEM, M - M199, S - supplements (FGF, EGF, insulin, ascorbic acid), VL - VascuLife.

For SMC isolation and expansion different culture media were used since a conclusive comparison of commonly described SMC culture media is lacking. To investigate the medium impact on SMC characteristics, marker expression of the cells was analysed after expansion in different media with 5 or 10% FCS as indicated in Table 1 and Supplementary Table S1. For flow cytometric surface marker analysis, single live cells were identified according to the gating strategy shown in Supplementary Fig. S2. Isolated cells were characterised as CD13^+^CD44^+^CD73^+^CD90^+^ and CD14^−^CD31^−^CD45^−^ (Fig. 2a). However, donor-dependent variability was detected regarding the expression of CD31 (ID1, ID2), CD45 (ID2), and CD90 (ID4), illustrating plasticity. No distinct pattern could be determined for the expression of CD140b (Platelet-derived growth factor receptor β), with half of the donors being negative (ID1, ID2, ID4) while for the other half (ID3, ID5, ID6) a certain expression was detectable.

**Table 1:** Cultivation media used for SMC isolation and expansion.

donor	expansion medium
ID1	VL
ID2	VL
ID3	D
ID4	D+S
ID5	M
ID6	M+S

D - DMEM, M - M199, S - supplements (FGF, EGF, insulin, ascorbic acid), VL - VascuLife.

Gene expression analysis of SMC marker demonstrated comparable level of *ACTA2*, *MYH11*, *MYOCD*, *TAGLN*, *PDGFRB*, *CNN1*, and *KCNMB1* expression in all isolated cells except those of ID5 (Fig. 2b). Overall, isolated cells show common SMC characteristics irrespective of the expansion medium used and FCS concentration. Nevertheless, expression of SMC marker might be impacted by the expansion medium used as indicated by the lack of MYH11 and TAGLN expression in cells isolated from ID5 which were expanded in M199. Along the same lines was the increased CD31 expression in cells derived from ID1 and ID2 which were cultivated in VascuLife.

### Contractility assessment indicates functional plasticity of SMC

**Figure 3:**
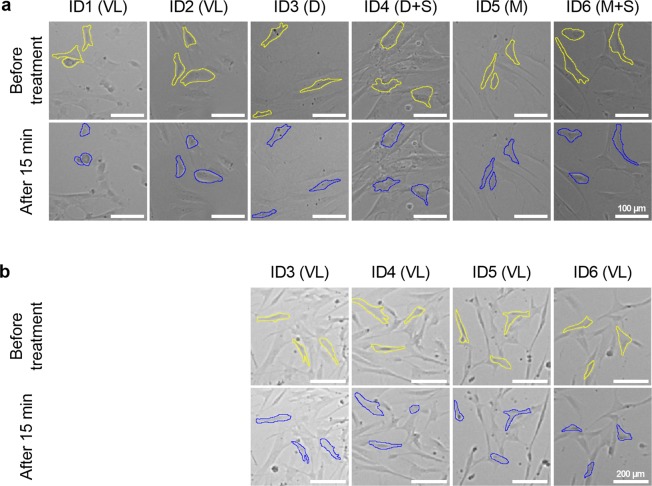
Contractility of SMC varies between donors. SMC expanded in different media were functionally assessed by stimulation with 10 µM carbachol for 15 min in (**a**) their respective expansion medium (given in brackets) or (**b**) after subcultivation in VascuLife medium for one passage. For visualization of stimulation-induced cell area change the membrane of single cells was marked; D - DMEM, M - M199, S - supplements (FGF, EGF, insulin, ascorbic acid), VL - VascuLife.

To further examine SMC characteristics, contractility of SMC was studied in a carbachol-based assay. Contraction of the isolated cells was induced by addition of carbachol to the expansion medium (Fig. 3a, Supplementary Movies a–f). To assess the impact of the medium, cells of ID3-6 were additionally tested after subcultivation in VascuLife medium for one passage (Fig. 3b, Supplementary Movies g–j). SMC derived from a healthy coronary artery (ID1) showed a distinct cell area reduction after 15 min while SMC derived from pathologically altered arteries showed no (ID3) or only slight contraction (ID2, ID4, ID6) in their respective expansion medium. Differences in contractility between the expansion medium and VascuLife medium could not be observed for ID3, ID4, and ID6, whereas an increased contractility after transfer to VascuLife medium was observed for ID5-derived SMC. Enhanced cell adhesion to the tissue culture plate in DMEM- and M199-based media might limit contractility of the SMC *in vitro*. Consequently, no distinct discrimination of SMC contractility depending on the expansion medium used could be determined. Collectively, functional assessment of SMC contractility illustrated donor-dependent differences and highlighted SMC plasticity.

### SMC proliferation varies between media

**Figure 4:**
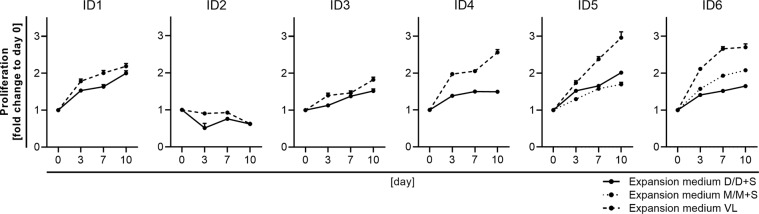
Proliferation of SMC varies between expansion media. SMC of passage three were seeded at 2 × 10^3^ cells/well and expanded in different media. Cell proliferation was determined at the indicated time points using the CyQuant Proliferation assay and is shown as the fold change to day zero; D - DMEM, M - M199, S - supplements (FGF, EGF, insulin, ascorbic acid), VL - VascuLife.

The proliferative capacity of the isolated cells expanded in different media was assessed to further determine donor-specific or medium-dependent differences. Except from SMC of ID2, all cells showed progressive proliferation independent of the culture medium (Fig. 4). Cells derived from ID2 were restricted in their proliferative capacity even after three weeks in culture compared to the cells derived from the other donors (expansion medium control pictures of ID2 in Fig. 7b). While proliferation rates between SMC cultures with 10% FCS in DMEM and M199 media were largely comparable, increased proliferation rates were observed in cultures with 5% FCS and VascuLife medium.

### Elevated alkaline phosphatase activity and phosphate level during osteogenic stimulation are indicative for an osteogenic differentiation of SMC

**Figure 5:**
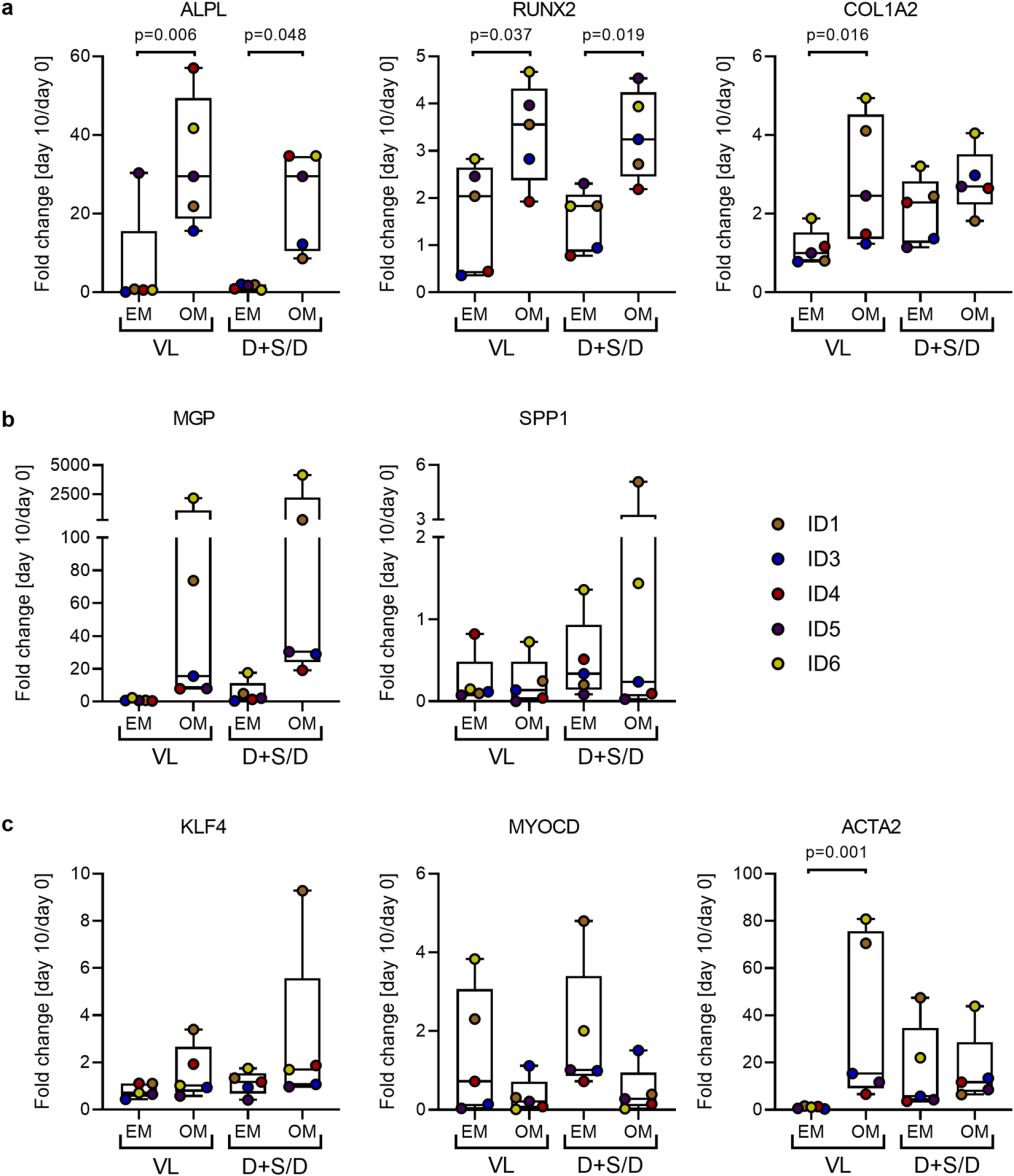
Osteogenic stimulation elevates alkaline phosphatase activity and phosphate level indicating an osteogenic differentiation of SMC. (**a**) Alkaline phosphatase (ALP) activity after osteogenic stimulation of SMC in VascuLife, DMEM, or M199. ALP activity was normalized to the metabolic activity of the cells determined with PrestoBlue Cell Viability Reagent. (**b**) Phosphate (PO4) level determined in the supernatant of SMC osteogenically stimulated in VascuLife, DMEM, or M199. ALP and PO4 values are given as mean of five replicate wells per donor and medium ± standard deviation; D - DMEM, M - M199, S - supplements (FGF, EGF, insulin, ascorbic acid), VL - VascuLife.

Since we found no direct dependence between the expansion medium used and the specific SMC characteristics, we wondered whether the culture medium affects the osteogenic differentiation potential of SMC. Therefore, SMC from each donor were osteogenically stimulated in their respective expansion medium supplemented with osteogenic additives. In addition, SMC were transferred to VascuLife and DMEM for osteogenic stimulation and corresponding expansion medium controls (Table 2). All osteogenic media were equally supplemented with FCS and glutamine in order to exclude an impact of these two factors on the osteogenic differentiation process. Alkaline phosphatase (ALP) activity, as an early indicator of osteogenic differentiation, was determined on day 0 (baseline) as well as day 4, 7, and 10 of the osteogenic stimulation. A constant increase in all three osteogenic differentiation media tested was detected (Fig. 5a) compared to the expansion medium controls (Supplementary Fig. S3a). Induction of ALP activity was slightly stronger after osteogenic stimulation in DMEM compared to VascuLife and comparable in DMEM and M199. Irrespective of the osteogenic medium used, ALP activity varied between donors, with ID1, ID2, and ID3 showing a strong upregulation while ALP activity remained weak until day ten in SMC cultures from ID4, ID5, and ID6. Interestingly, baseline ALP activity at day zero was especially high in SMC derived from a healthy coronary artery (ID1), upregulation of its activity plateaued at day seven. In contrast, SMC derived from pathologically altered arteries of different arterial origin (ID2-6) showed lower baseline ALP activity and its upregulation did not reach a plateau until day ten.

**Table 2:** Osteogenic media and expansion medium controls used for comparison regarding the differentiation potential of SMC.

donor	expansion medium control (EM)					osteogenic medium (OM)		
	VL	D	D+S	M	M+S	VL	D	M
ID1	x	x				x	x	
ID2	x	x				x	x	
ID3	x	x				x	x	
ID4	x		x			x	x	
ID5	x	x		x		x	x	x
ID6	x	x			x	x	x	x

EM - expansion medium; OM - osteogenic medium (EM with 50 µM ascorbic acid, 10 mM β-Glycerophosphate and 0.1 µM dexamethasone); D - DMEM, M - M199, S - supplements (FGF, EGF, insulin, ascorbic acid), VL - VascuLife.

In addition to the ALP activity, the concentration of its direct metabolite phosphate (PO4), was determined in the supernatant throughout the differentiation process. The increase of ALP activity in all osteogenically stimulated SMC correlated with an increase of PO4 (Fig. 5b) compared to the expansion medium controls (Supplementary Fig. S3b). Elevated ALP activity of SMC osteogenically stimulated in DMEM correlated with higher phosphate level in these cultures compared to corresponding cultures in VascuLife. Although ALP activity of osteogenically stimulated SMC was comparable in DMEM and M199, phosphate level remained lower in M199-stimulated cultures from day 14 onwards. In line with the elevated ALP activity in osteogenically stimulated SMC derived from a healthy coronary artery (ID1), PO4 level were distinctly increased compared to SMC cultures derived from pathologically altered arteries (ID2-6). Regardless of the osteogenic differentiation medium used and the induced ALP activity, SMC from pathologically altered arteries (ID2-6) showed an early and sharp increase in phosphate levels within the first four days and remained at that plateau thereafter. In contrast, phosphate levels of SMC cultures derived from a healthy coronary artery (ID1) increased until day 14, before reaching a plateau. For the differentiation of these SMC in DMEM, a distinct drop of the phosphate level was observed after day 14, indicating enhanced deposition of calcium-phosphate matrix and thus withdrawal of phosphate from the supernatant. An early and ongoing decrease in phosphate level after osteogenic stimulation in DMEM was also noted for SMC from ID4. Taken together, elevated ALP activity during osteogenic stimulation indicated an osteogenic differentiation of SMC in all media tested, but is most distinct after osteogenic stimulation in DMEM. Upregulation of ALP activity resulted in elevated phosphate level while a decrease of the latter seems to correlate with calcification of SMC *in vitro*.

### Increased gene expression of osteogenic markers substantiates an osteogenic differentiation while SMC characteristics are partially maintained

**Figure 6:**
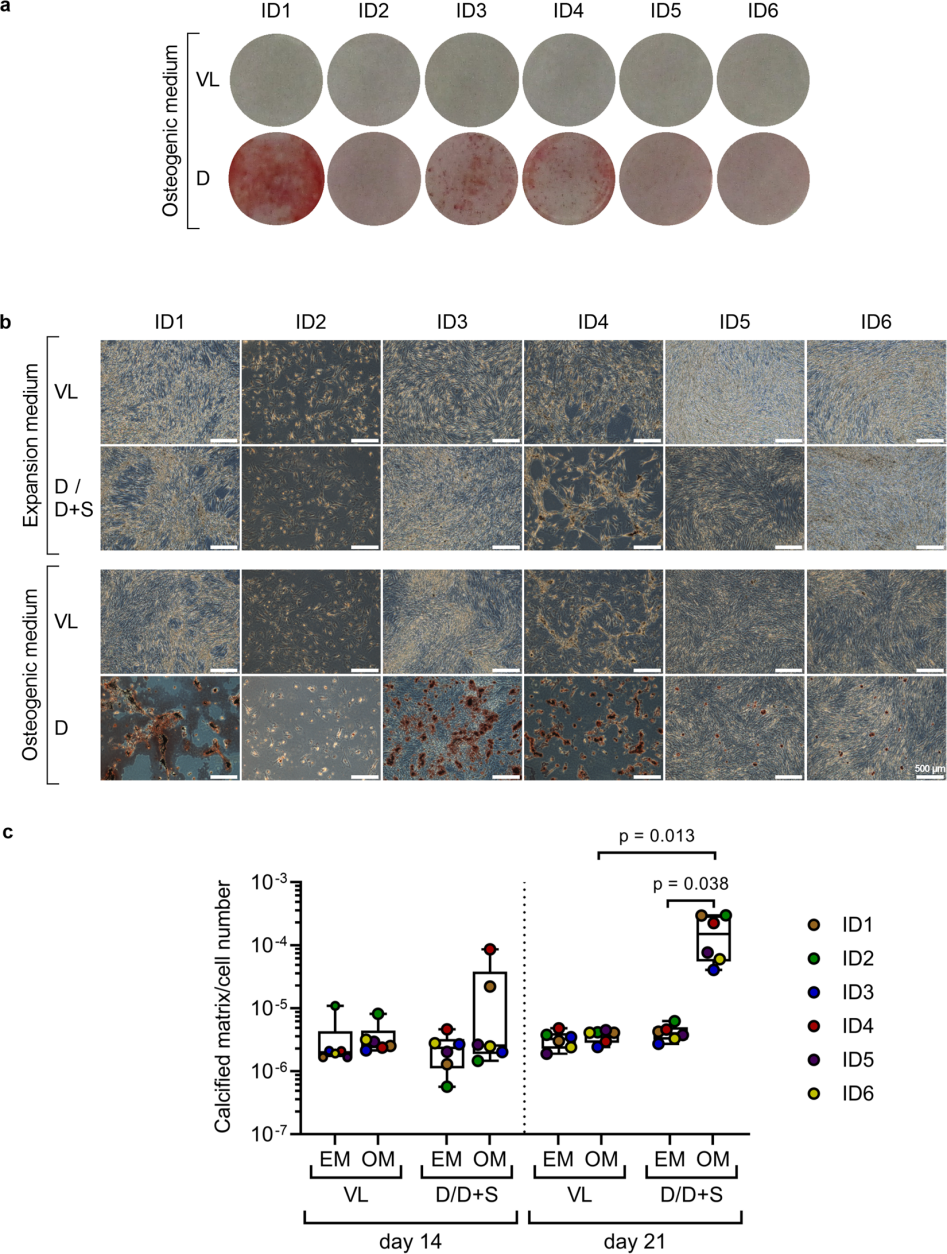
Increased gene expression of osteogenic markers after stimulation in VascuLife and DMEM substantiates an osteogenic differentiation of SMC while SMC characteristics are partially maintained. Gene expression analysis of SMC osteogenically stimulated in VascuLife or DMEM for ten days; (**a**) osteogenic marker, (**b**) negative regulators of calcification, (**c**) SMC marker and transcriptional regulators. Expression was normalized to the expression of the housekeeping gene *RPL13A* and the fold change between day zero (baseline) and day ten was calculated. Expansion medium controls for DMEM and DMEM plus supplements were pooled for quantitative analysis. Gene expression analysis of ID2-derived SMC was not possible due to low cell numbers and resulting insufficient amounts of isolated mRNA. For statistical analysis a Kruskal-Wallis-test with an uncorrected Dunn’s post test was used. Only significant differences (p < 0.05) are given; D - DMEM, M - M199, S - supplements (FGF, EGF, insulin, ascorbic acid), VL - VascuLife, EM - expansion medium, OM - osteogenic medium.

To further investigate the osteogenic differentiation in SMC, expression of surrogate marker genes for the osteogenic differentiation, negative regulation of calcification and SMC lineage were analysed at day ten of the osteogenic stimulation and compared to baseline level at day zero. A distinct increase of *ALPL* and the pro-osteogenic transcription factor *RUNX2* could be observed for all SMC after ten days of osteogenic stimulation in all media tested (Fig. 6a, Supplementary Fig. S4a) compared to the respective expansion medium controls (EM vs. OM: *ALPL* (VL) p = 0.006; *ALPL* (D) p = 0.048; *RUNX2* (VL) p = 0.037; *RUNX2* (D) p = 0.019; for osteogenic stimulation in M199 no statistical analysis was possible). For *COL1A2* a marked increase was only detected after osteogenic stimulation in VascuLife (EM vs. OM: *COL1A2* (VL) p = 0.016) and M199 whereas only a slight upregulation was seen after osteogenic stimulation in DMEM. However, cultivation in DMEM already increased the constitutive expression of *COL1A2* as detected in the expansion medium controls (Fig. 6a, Supplementary Fig. S4a). This general increase in *COL1A2* expression might favour deposition of calcified matrix.

**Figure 7:**
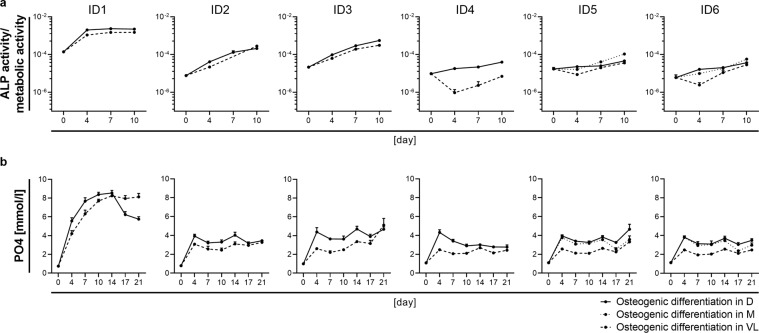
SMC deposit calcified matrix only after osteogenic stimulation in DMEM. Alizarin red staining of calcified matrix deposited by SMC osteogenically stimulated in different culture media for 14 and 21 days. (**a**) Macroscopic examination of calcified matrix deposition by SMC after 21 days of osteogenic stimulation in VascuLife or DMEM. (**b**) Microscopic examination of the wells and their respective expansion media controls after 21 days of osteogenic stimulation in VascuLife or DMEM. (**c**) Quantification of the deposited calcified matrix normalized to the cell number on day 14 and day 21. Expansion medium controls for DMEM and DMEM plus supplements were pooled for quantitative analysis. Values of the individual donors are shown as points superimposed onto the boxplots and are given as mean of five replicate wells per donor and medium. For statistical analysis data of each point in time were compared amongst each other using a Kruskal-Wallis-test with a Dunn’s post test corrected for multiple comparisons. Only significant differences (p < 0.05) are given; D - DMEM, S - supplements (FGF, EGF, insulin, ascorbic acid), VL - VascuLife, EM - expansion medium, OM - osteogenic medium.

Independent from the culture medium, expression of *MGP*, a calcification inhibitor that prevents mineral precipitation in the extracellular matrix^35^, was markedly increased after osteogenic stimulation (Fig. 6b, Supplementary Fig. S4b). In contrast, expression of *SPP1*, an inhibitor of ectopic calcification^35^, remained weak in all osteogenically stimulated cultures and expansion medium controls. Except for the cultures of ID1 and ID6, expression was slightly downregulated during the osteogenic differentiation process of SMC.

In line with the increased expression of osteogenic markers krüppel-like factor 4 (*KLF4*), a stem cell factor and transcriptional repressor of SMC specific genes^36^, is slightly increased in osteogenically stimulated SMC while *MYOCD*, a transcription factor promoting the SMC lineage^9^, is downregulated (Fig. 6c, Supplementary Fig. S4c). Interestingly, *ACTA2*, characteristic for the SMC phenotype, was upregulated in correlation to the osteogenic markers (EM vs. OM: *ACTA2* (VL) p = 0.001; Fig. 6c, Supplementary Fig. S4c). Collectively, gene expression analysis of osteogenic markers further confirmed an osteogenic differentiation while SMC characteristics are partially maintained.

### SMC deposit calcified matrix only after osteogenic stimulation in DMEM

To confirm the osteogenic differentiation of SMC in different cultivation media, deposition of calcified matrix was analysed by alizarin red staining. After 21 days deposition of calcified matrix could only be detected in those cultures osteogenically stimulated in DMEM (Fig. 7a,b, Supplementary Fig. S5a,b). This effect was independent of the expansion medium used before differentiation in DMEM. Nevertheless, conditioning effects of the expansion medium seem to impair the osteogenic differentiation in DMEM as shown for SMC of ID5 and ID6, both expanded in M199-based medium. Quantification of the deposited calcified matrix normalized to the cell number confirmed the significant difference between the osteogenic media tested (OM (D) vs. OM (VL) after 21 days of osteogenic stimulation: p = 0.013; Fig. 7c, Supplementary Fig. S5c).

Staining intensity and differences in the quantified amount of deposited calcified matrix depict donor-dependent differences in calcification onset and capacity (Fig. 7b,c, Supplementary Fig. S5b,c) with SMC derived from a healthy coronary artery (ID1) and SMC derived from a carotis stenosis (ID4) showing distinct deposition of calcified matrix already on day 14 of osteogenic stimulation in DMEM. SMC of ID4 even showed contraction of the cell layer and minor deposition of calcified matrix in the D+S expansion medium control, indicating a preosteoblastic imprint of these cells from the previous pathologic alterations in the A. carotis. For the donors ID1 and ID4 a drop or decrease in the supernatant phosphate level was noted that correlates with the amount of deposited calcified matrix (Fig. 6b). All other SMC started to deposit increasing amounts of calcified matrix only after day 14, suggesting the need of a prolonged osteogenic stimulus before a substantial osteogenic differentiation in those SMC is induced that results in deposition of calcified matrix. Overall, osteogenic differentiation confirmed the potential of SMC to deposit calcified matrix, for this stimulation in DMEM is required.

Although only SMC cultures stimulated in DMEM deposited calcified matrix, proliferation and cell numbers were distinctly lower in all cultures with osteogenic media compared to their corresponding expansion medium controls (Supplementary Fig. S6), indicating successful induction of a switch from proliferation towards differentiation. No significant differences in cell numbers between the different osteogenic media and expansion media controls or between day 14 and day 21 could be determined. However, SMC of ID3 show an increased proliferation in response to the osteogenic stimulus. This is in line with an increased expression of the transcription factor *MYOCD* in these cultures on day 10 (Fig. 6c) whereas all other SMC cultures show a reduced expression of *MYOCD* under the osteogenic stimulus.

## Discussion

**Figure 8:**
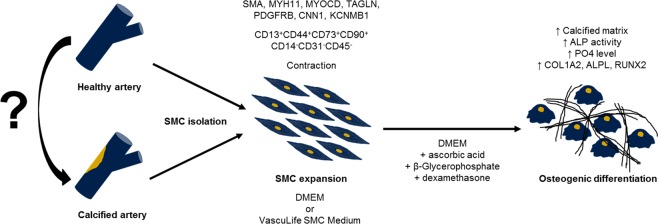
Schematic work flow for the *in vitro* analysis of vascular calcification.

In this study, we established and optimized a human *in vitro* model for the investigation of advanced atherosclerosis and vascular calcification, based on SMC derived from healthy and diseased donors. The model allows to study late-stage intervention strategies for patients with already established disease and calcified plaques and thus addresses an increasing clinical need in aging societies. Although, *in vitro* cultivation of SMC might be limited in mimicking disease-relevant *in vivo* processes^11,37,38^, our model with its comprehensive work flow can be used to investigate donor-dependent differences of potential therapeutic relevance as well as broader screening for potential pathologic mediators causing vascular calcification (Fig. 8). Thus, being of advantage compared to animal models used in other studies.

Our comprehensive characterisation of the isolated SMC, together with the functional evaluation, considers SMC plasticity and thus adds weight to previously described *in vitro* models^20,21,23–25^. Furthermore, a systematic comparison of commonly described culture media optimizes the conditions to properly investigate the osteogenic differentiation potential of SMC, including potential pathogenic mediators. The combined approach of digestion and explant culture was feasible for the isolation of SMC from all patient-derived samples which were obtained from different arterial locations and had different degrees of calcification. Irrespective of the culture medium used and its FCS concentration all isolated cells exhibited an SMC-like phenotype. However, isolation protocols have to consider broadly described SMC plasticity^1,9^. In that regard, it has to be assumed that the obtained SMC are the more viable and migratory ones. Although, potential donor-dependent differences might have vanished as an effect of the isolation protocol and the culture conditions^38^, flow cytometric surface marker analysis characterised all SMC as CD13^+^CD44^+^CD73^+^CD90^+^ and CD14^−^CD31^−^CD45^−^. Except for the expression of CD90, this marker pattern is in line with the study by Kumar *et al*. (2017) characterising SMC derived from human mesenchymoangioblast^39^. Of these, CD44 and CD90 were recently described as identifier for a relatively rare population of mesenchymal progenitor cells within the vessel wall^40–42^. However, except for SMC of ID4, derived from a carotis stenosis, all our SMC, including those from a healthy coronary artery, showed CD44 and CD90 expression of the entire cell population. This suggests that both markers are commonly expressed by arterial SMC while expressional changes might rather be an effect of phenotypic transition^12^. Since an impact of CD90 for the osteogenic differentiation of mesenchymal stromal cells was demonstrated recently^43–45^, its expression on SMC might also be of relevance considering their potential for osteogenic differentiation.

Further variability, highlighting donor-dependent differences and SMC plasticity, was observed for the expression of CD31, CD45, and CD140b. CD31 expression might be indicative of an immature SMC phenotype^39^. However, its induction seems to depend on the cultivation medium used, since only SMC cultivated in VascuLife showed a tendency to express CD31. Expression of CD45 in SMC derived from ID2 might indicate that those cells are descendants of an endothelial to mesenchymal transition occurred during lesion formation as described for mitral valve endothelial cells after myocardial infarction^46^. Variance concerning the expression of CD140b could only be observed when measured as surface protein, not in the gene expression analysis. This discrepancy might emerge from regulated trafficking of CD140b^47^ that seems to be of relevance during SMC recruitment to the fibrous cap^14,48^ and further illustrates SMC plasticity.

Moreover, isolated cells from pathologically altered arterial samples and SMC derived from a healthy coronary artery showed a similar expression of the SMC markers *ACTA2*, *MYH11*, *MYOCD*, *TAGLN*, *PDGFRB*, *CNN1*, and *KCNMB1*. This expression pattern characterises the cells as SMC, albeit, not all of these markers are considered as exclusive for the SMC lineage and expression might change due to phenotypic plasticity. Yet, expression of *KCNMB1*, a beta-1 subunit of calcium-activated potassium channels involved in vasoregulation, was shown to be restricted to vascular smooth muscle tissue^49,50^. Furthermore, *MYH11* is considered as the most discriminating marker of the SMC lineage^9,11^, its detection in the immunofluorescence staining confirmed the SMC phenotype. Generally, only low expression levels of genes associated with contractility seem to be required to maintain a certain protein level in SMC, possibly reflecting the low turnover of contractile proteins^38,51^. Thus, low mRNA expression levels of *MYH11*, but distinct protein levels as assessed by immunofluorescence staining confirms the low turnover of this contractile protein even after passaging *in vitro*.

Besides the presence of contractile filaments, functional assessment of the isolated cells using a contraction assay highlighted SMC plasticity. Observed differences in contractility between SMC cultures derived from different donors might be dependent on the cytoskeleton arrangement possibly affected by the culture conditions. Earlier studies showed that *in vitro* cultivation of SMC could induce a phenotypic transition from a contractile phenotype towards a synthetic state with diminished contractility^8–10^. In that regard, diminished serum concentrations as in VascuLife medium seem to favour a contractile SMC phenotype by inhibiting modulation towards a proliferative state^10^. We observed no distinct difference in SMC ability to contract between the initial expansion medium or after subcultivation in VascuLife medium for one passage. Nevertheless, an impact of the medium used for cell isolation and initial expansion on contractility cannot be fully excluded in later passages, especially since medium-dependent differences in cell proliferation were observed that might limit contractility.

Overall, although the embryological origin of the SMC isolated in our study might vary according to the location they are derived from^52,53^, we could not determine distinct differences of the SMC characteristics dependent on the location of sample removal, clinical background, or the expansion medium used. However, with regard to the subsequent osteogenic stimulation and the potential for deposition of calcified matrix, SMC expansion in either VascuLife or DMEM is recommended. Due to the limited number of donors in our study, broader screening with additional donors might be needed to enable further discrimination.

In line with the similar SMC characteristics determined, all SMC exhibited a similar osteogenic differentiation potential, irrespective of their arterial location and their embryological origin^52,53^. However, even after adjusting FCS and glutamine concentrations, the degree of osteogenic differentiation and deposition of calcified matrix was strongly dependent on the cultivation medium used, illustrating the importance of environmental cues for the behaviour of SMC^9^. Irrespective of the expansion medium used, differentiation including substantial deposition of calcified matrix was only observed after osteogenic stimulation in DMEM. Considering calcification onset and capacity, donor-dependent differences were notified, with some donors requiring a prolonged osteogenic stimulus of 21 days until a substantial deposition of calcified matrix could be observed, whereas others showed substantial matrix deposition already on day 14. A preosteoblastic imprint of SMC from ID4, reflected by contraction of the cell layer even in the expansion medium control, might have triggered an enhanced osteogenic differentiation in those SMC. Furthermore, decreased CD90 expression, as detected in SMC from this donor, might be indicative for a matured osteoblastic transition^44,54^. Enhanced osteogenic differentiation of SMC derived from a healthy coronary artery, might result from an increased susceptibility to *in vitro* stimulation. Since cells derived from a healthy donor are exposed to differentiation stimuli for the first time in our *in vitro* model, they seem to be more prone to respond to the stimulation compared to SMC derived from pathologically altered arteries. The latter might have adjusted to their previous disease state, thus requiring a prolonged stimulation *in vitro* to undergo further phenotypic modulation and differentiation.

In five out of six SMC cultures the observed osteogenic differentiation was accompanied by reduced proliferation and cell numbers. Ongoing proliferation was only observed for SMC from ID3 and is in line with an increased *MYOCD* expression while all other SMC cultures showed a reduced expression of *MYOCD* under the osteogenic stimulus. Increased proliferation of SMC from ID3 might thus be driven by a sustained expression of the transcription factor *MYOCD*.

Besides the medium composition, calcium and phosphate levels are highly relevant for the *in vitro* calcification of SMC. Increased calcium and phosphate concentrations were shown to induce SMC calcification, thus, their ratio should be considered during stimulation to avoid side effects from additional stimulation, e.g. earlier calcification with elevated calcium level, and supplementary signalling^1,29,55–58^. In our approach all cultivation media have a similar calcium concentration while the basal phosphate concentration is slightly lower in VascuLife medium compared to DMEM and M199 (Supplementary Table S1). For the osteogenic stimulation media were supplemented with β-Glycerophosphate disodium salt hydrate, an organic phosphate donor that requires enzymatic activity of alkaline phosphatase to increase the extracellular phosphate concentration above baseline level. Such active cell involvement in the generation of inorganic phosphate occurs more suitable for the study of triggers and mechanisms involved in the osteogenic differentiation of SMC than supplementing media with inorganic phosphate and calcium that could be disease-relevant triggers themselves^55,56,59,60^.

In our model increased ALP activity was reflected by increased *ALPL* gene expression. Together with an upregulated expression of the osteogenic markers *RUNX2* and *COL1A2* an osteogenic differentiation of the SMC was substantiated that is likely to be mediated by the induction of the stem cell factor and transcriptional repressor of SMC specific genes *KLF4*^36^. Parallel to the upregulation of osteogenic differentiation markers upon stimulation and in line with an earlier *in vitro* study investigating calcification of human vascular cells^61^, expression of *MGP*, a negative regulator of mineral precipitation^28,35^, was markedly increased after ten days of osteogenic differentiation. Elevated *MGP* expression might be indicative for a counteracting strategy of the SMC while being exposed to a sustained osteogenic stimulus. However, the synthesised MGP might be under-carboxylated and thus dysfunctional as mineralization inhibitor^62^. Compared to *MGP*, expression of *SPP1*, another known negative regulator of calcification^35^, remained weak. Such diminished gene expression and very low concentrations of secreted osteopontin protein in human SMC cultures were described earlier^61^. Although osteopontin is described to be an inducible inhibitor of vascular calcification *in vivo*^63^, SMC were shown not to be the predominant source during vascular calcification^61^. Considering the phenotypic switching and osteogenic differentiation of SMC, previous studies furthermore indicated that this process is accompanied by a loss of SMC markers like *ACTA2*, *TAGLN*, *MYH11*, and *CNN1*^1,20,36^. Although *MYOCD* as a potent inducer of SMC marker genes^9^ is downregulated after osteogenic stimulation in all SMC cultures except those of ID3, we detected a distinct increase of *ACTA2* expression, indicating that despite an osteogenic differentiation the SMC phenotype is still partially maintained^53^. In contrast to the above mentioned studies, such co-expression of osteogenic and SMC marker was demonstrated earlier *in vitro* and *ex vivo*^5,19,61^. Increased expression of *ACTA2* might thereby be induced upon growth arrest, e.g. at high cell densities^64^. However, the outlined contradictions will require further investigations.

Overall, our in depth study of SMC from six human donors highlights their phenotypic plasticity and their dependency on complex interactions of local environmental cues. In that regard, we could demonstrate that vascular calcification *in vitro* is mimicked best by the osteogenic differentiation of SMC in DMEM. With our human SMC-based, disease-relevant *in vitro* model donor differences and disease-associated imprints can be detected that can be of relevance for personalized treatment. Furthermore, broader screening for potential pathogenic mediators that cause vascular calcification and, considering the 3R approach, translational oriented studies to identify and evaluate therapeutic targets are possible by addition of patient sera, cytokines, drugs, inhibitors or co-culture approaches.

## Methods

### Sample acquisition and ethics statement

Total endarterectomy samples of calcified arteries and aneurysms (Fig. 1) from different arterial locations were obtained from the University Clinic Ruhr, Bochum, Germany according to the Declaration of Helsinki after ethical approval of the Ethics Committee, Medical Faculty of Ruhr-University Bochum (Registration-Number: 16-5714, 07.07.2016). Samples were stored at 4 °C either in serum free Dulbecco’s Modified Eagle Medium low glucose (D5546; DMEM), M199 (M4530, both Sigma), Ringer’s solution (Fresenius) or Hank’s balanced salt solution with sodium bicarbonate, supplemented with 0.025 M HEPES (both Sigma) and 1% penicillin/streptomycin (Biochrom) until SMC isolation. Commercially available healthy coronary artery SMC (Lifeline Cell Technology) were used for comparison.

### SMC isolation and expansion

SMC were isolated combining digestion and explant culture. Samples were rinsed in phosphate buffered saline (PBS, Gibco) and remaining endothelium was scrapped off with a scalpel where possible. Heavily calcified areas, fat tissue, and adventitial remnants were removed to dissect intimal and medial fragments. Tissue fragments were rinsed again in PBS, minced, and enzymatically digested in 2 mg/ml collagenase I (Gibco) in serum-free medium for 3 to 4 hours until the tissue was well loosened. For isolation and expansion several cultivation media described as suitable for SMC cultures were compared. Accordingly, cell suspension and remaining tissue fragments were placed in cell culture flasks with either VascuLife SMC Medium (VL; CellSystems GmbH), a 5% FCS medium, DMEM (D) supplemented with 1% Glutamax (Invitrogen), or M199 (M), the latter two being further supplemented with 1% penicillin/streptomycin and 20% fetal calf serum (FCS; Biochrom) during passage zero or 10% FCS from passage one on. For additional comparison, DMEM and M199 were supplemented with the same additives as used in VascuLife SMC Medium, that is 50 µg/ml L-Ascorbic acid 2-phosphate sesquimagnesium salt hydrate, 5 µg/ml insulin (both Sigma-Aldrich), 5 ng/ml endothelial growth factor (EGF; BioLegend), and 5 ng/ml recombinant human fibroblast growth factor (FGF; PeproTech; DMEM plus supplements (D+S); M199 plus supplements (M+S)). An overview of the cultivation media composition is given in Supplementary Table S1. Sample size limited isolation and primary cell expansion to one cultivation medium per donor (Table 1). Medium was exchanged one day post seeding and twice a week thereafter. Cells were cultivated until near confluency; tissue fragments remained in the flask until the end of passage zero.

### Immunofluorescence

SMC of passage three and human skeletal muscle myoblasts (Lonza) were grown in 96-well plates (Falcon), fixated with Roti-Histofix 4% (Roth), permeabilized with 0.2% Triton X-100 (Sigma) in PBS, and blocked with 1% bovine serum albumin (BSA; Sigma) and 5% normal goat serum (NGS; Vector Laboratories) in PBS. Each step was performed for 10 min. Antibodies and dilutions used for staining are summarized in Table 3. Cells were incubated over night at 4 °C with the respective primary antibody diluted in PBS with 1% BSA. The secondary antibody was diluted in PBS with 2% BSA and 2% NGS and cells were incubated for 1 h at room temperature. Nuclei were visualized with 1 µg/ml 4′,6-Diamidin-2-phenylindol (DAPI; Sigma) diluted in PBS for 10 min at room temperature. Images of immunolabelled cells were acquired using a Leica DMI 6000B with a 20× HCX PL FLUOTAR objective, equipped with a Leica DFC345 FX camera and the imaging software Leica Application Suite V2.5.0.6735 (Leica Microsystems CMS GmbH).

**Table 3:** List of antibodies used for SMC immunofluorescence and flow cytometry.

Antibody target	Clone	Conjugate	Dilution	Application	Company	Catalogue number (Lot)
Anti-human SMA	1A4	PE	1/50	IF	R&D Systems	IC1420P (B174246)
Anti-human MYH4	MF20	—	1/250	IF	eBioscience	14-6503-82 (CAEI0818101)
Anti-human MYH11	1G12	—	1/100	IF	Abcam	ab683 (GR273859-7)
Goat anti-mouse IgG (H + L)	Polyclonal	Alexa Fluor 488	1/400	IF	Thermo Fisher Scientific	A-11001 (1572559)
Anti-human CD13	WM15	APC-R700	1/400	FC	BD Horizon	565124 (7353767)
Anti-human CD14	MfP9	FITC	1/200	FC	BD	345784 (13599)
Anti-human CD31	WM59	PE	1/100	FC	BD Pharmingen	555446 (16944)
Anti-human CD44	IM7	BV785	1/100	FC	BioLegend	103041 (B206615)
Anti-human CD45	HI30	BV510	1/100	FC	BioLegend	368525 (B222302)
Anti-human CD73	AD2	BV605	1/50	FC	BD Horizon	563199 (7026510)
Anti-human CD90	5E10	APC	1/800	FC	BioLegend	328114 (B174246)
Anti-human CD140b	28D4	PerCP/Cy5.5	1/50	FC	BD Pharmingen	562714 (7083965 & 722771)

If not stated otherwise, all antibodies are monoclonal; IF - immunofluorescence, FC - flow cytometry.

### Quantitative real-time PCR

For gene expression analysis, mRNA was isolated using the RNeasy Plus mini kit (Qiagen) and mRNA concentration was determined using a NanoDrop ND-1000 spectrophotometer (PeqLab). 250 ng mRNA were reverse transcribed to cDNA using the iScript cDNA Synthesis Kit (Bio-Rad). Quantitative real-time PCR (qRT-PCR) was performed using the SYBR Green PCR master mix (Roche) and the primer pairs summarized in Table 4. Reactions were run on a LightCycler 480 II (Roche). For SMC characterisation cells at passage one or two were used and relative expression levels were calculated by normalizing minimal cycle threshold values (Ct) to the respective expression of the housekeeping gene *RPL13A*. To analyse the gene expression during osteogenic differentiation the fold change of each gene of interest was calculated, comparing the expression at day ten in the respective differentiation medium to the expression at day zero in the respective expansion medium of each donor. No mRNA analysis during osteogenic differentiation of SMC from ID2 was possible due to insufficient cell numbers.

**Table 4:** List of primers used for qRT-PCR.

Gene	Abbre-viation	Direction	Sequences 5′ → 3′	Reference ID
Alkaline phosphatase, tissue-nonspecific	*ALPL*	Forward	ATGTTCCTGGGAGATGGGATG	—
		Reverse	ACCTGGGCATTGGTGTTGTA	
Aortic smooth muscle actin, alpha2	*ACTA2*	Forward	GCCAAGCACTGTCAGGAATC	—
		Reverse	GGGTACTTCAGGGTCAGGAT	
calcium-activated potassium channel subunit beta-1	*KCNMB1*	Forward	GAGAAGGTCAGAGCCAAATTCC	25777641c2
		Reverse	AATAGGACGCTGGTTTCGTTC	
Calponin 1	*CNN1*	Forward	GAACGTGGGAGTGAAGTACGC	56676373c3
		Reverse	CAGCCCAATGATGTTCCGC	
Collagen type I alpha 2 chain	*COL1A2*	Forward	AGCCGGAGATAGAGGACCAC	—
		Reverse	GGCCAAGTCCAACTCCTTTT	
Krüppel-like factor 4	*KLF4*	Forward	CGGACATCAACGACGTGAG	194248076c3
		Reverse	GACGCCTTCAGCACGAACT	
Matrix gla protein	*MGP*	Forward	TCCGAGAACGCTCTAAGCCT	299890878c1
		Reverse	GCAAAGTCTGTAGTCATCACAGG	
Myocardin	*MYOCD*	Forward	ACGGATGCTTTTGCCTTTGAA	226423888c1
		Reverse	AACCTGTCGAAGGGGTATCTG	
Myosin heavy chain 11	*MYH11*	Forward	GGTCACGGTTGGGAAAGATGA	92091584c2
		Reverse	GGGCAGGTGTTTATAGGGGTT	
Osteopontin	*SPP1*	Forward	CACTACCATGAGAATTGCAGTGA	—
		Reverse	CTGCTTTTCCTCAGAACTTCCA	
Platelet-derived growth factor receptor β	*PDGFRB*	Forward	TCTTTGTGCCAGATCCCACC	—
		Reverse	AGTGCAACGTCCCCTTTCTT	
Ribosomal protein L13a	*RPL13A*	Forward	CCTGGAGGAGAAGAGGAAAGAGA	—
		Reverse	TTGAGGACCTCTGTGTATTTGTCAA	
Runt related transcription factor 2	*RUNX2*	Forward	CTCCTACCTGAGCCAGATGA	—
		Reverse	CGGGGTGTAAGTAAAGGTGG	
Transgelin	*TAGLN*	Forward	GAAACCCACCCTCTCAGTCA	—
		Reverse	ATGTCTGGGGAAAGCTCCT	

Primer were designed using NCBI Primer-BLAST or primers from the Harvard PrimerBank (pga.mgh.harvard.edu/primerbank) were used; respective reference IDs are given for the latter.

### Flow cytometry analysis

Single cell suspensions of passage three or four were prepared using 0.05% Trypsin-EDTA (Gibco). To avoid unspecific antibody binding to Fc receptors (FcR), cells were blocked with FcR Blocking Reagent (Miltenyi Biotech) according to the manufacturer’s instruction. Cell surface marker were stained for 10 min using the antibodies and dilutions given in Table 3. LIVE/DEAD^TM^ Fixable Blue Dead Cell Stain Kit (Invitrogen) was used to exclude dead cells. Cells were acquired using a CytoFLEX LX flow cytometer (Beckman Coulter). Flow cytometric data were analysed with FlowJo V10 (FlowJo, LLC).

### Contraction assay

SMC cultures were stimulated with 10 µM carbachol (Sigma) in their respective expansion medium. To further assess the medium impact on cell contractility, cells of ID3-6 were additionally transferred to VascuLife medium for one passage before being stimulated with carbachol in this medium. Time lapse images were recorded for 15 min at 37 °C and 5% CO_2_ using a Leica DMI 6000B equipped with a Leica DFC345 FX camera and a BL-X incubator (Leica Microsystems CMS GmbH). Images were acquired using a 5× or 10× HCX PL FLUOTAR objective and the imaging software Leica Application Suite V2.5.0.6735. Time lapse serial images were converted to QuickTime movies and the membrane of single cells was marked for visualization of stimulation-induced cell area change using ImageJ software (NIH).

### Proliferation assay

SMC at passage three were seeded in 96-well plates with 2 × 10^3^ cells/well and 64 µl medium/well; five replicate wells per donor and expansion medium were seeded. To compare medium effects on cell proliferation, 24 h post-seeding (baseline day zero) expansion medium of each SMC culture was replaced by the expansion media given in Table 2. On day zero, three, seven, and ten wells were washed with PBS and stored empty at −80 °C until further analysis. Cell count was determined using the CyQuant proliferation assay. The fold change to day zero was calculated for each time point and cell numbers are given as mean of five replicate wells per donor and medium ± standard deviation.

### Osteogenic differentiation of SMC

Osteogenic differentiation of SMC at passage four was performed in 96-well plates with 2 × 10^3^ cells/well and 64 µl medium/well; five replicate wells per donor and expansion medium control or osteogenic stimulation were seeded. For mRNA isolation SMC were seeded in 6-well plates with 6×10^4^ cells/well and 2 ml medium/well; three replicate wells per donor and expansion medium control or osteogenic stimulation were pooled for mRNA isolation. All SMC were seeded in their respective expansion medium.

24 h post-seeding, osteogenic differentiation of SMC was induced. To assess the medium impact on their differentiation potential, SMC from each donor were on the one hand osteogenically stimulated in their respective expansion medium while, on the other hand, expansion and osteogenic medium (EM; OM) were changed to either VascuLife or DMEM (Table 2). For the osteogenic differentiation each medium was equally supplemented with 10% FCS, 1% Glutamax and 1% penicillin/streptomycin, 50 µM L-Ascorbic acid 2-phosphate sesquimagnesium salt hydrate, 10 mM β-Glycerophosphate disodium salt hydrate and 0.1 µM water soluble dexamethasone (the last three all Sigma-Aldrich) according to a standard protocol used for the differentiation of mesenchymal stromal cells^65^. SMC were osteogenically stimulated for 21 days, with medium exchange every 3 to 4 days. Cell culture supernatant was collected for phosphate analysis at each medium exchange and stored at −80 °C until further analysis.

### Evaluation of metabolic activity

Metabolic activity of the cells was assessed using the PrestoBlue Cell Viability Reagent (Invitrogen). Ten volumes of pre-warmed expansion medium were mixed with one volume of PrestoBlue Cell Viability Reagent. Cells stimulated in 96-well plates were incubated with 64 µl of this mixture for 1 h at 37 °C and 5% CO_2_. Absorption at 590 nm was measured using an infinite M200PRO plate reader (Tecan); blank values were subtracted.

### Evaluation of alkaline phosphatase activity

The enzymatic activity of alkaline phosphatase (ALP) was assessed at day 0, 4, 7, and 10 by its turnover of para-nitrophenyl phosphate disodium hexahydrate (*p*NPP; Sigma) as described previously^66^. The volume of all solutions was adjusted as follows. Wells were rinsed with 150 µl PBS, followed by a wash with 200 µl AP-buffer. SMC were then incubated with 100 µl of *p*NPP substrate mixed 1:1 with AP-buffer for 10 min at 37 °C and 5% CO_2_, the reaction was terminated by addition of an equal volume of 1 M sodium hydroxide solution (Sigma). 100 µl were transferred to a 96-well plate and absorption was measured at 405 nm using an infinite M200PRO plate reader. Calculated ALP activity was normalized to the prior determined metabolic activity of the cells and is given as mean of five replicate wells per donor and medium control ± standard deviation.

### Phosphate assay

Phosphate (PO_4_) concentrations were determined in the supernatant of each well for each media exchange using the Phosphate Assay Kit (Abcam) according to the manufacturer’s instructions. Absorbance was measured at 650 nm using an infinite M200PRO plate reader; blank values were subtracted. Values are given as mean of five replicate wells per donor and expansion medium control or osteogenic stimulation ± standard deviation.

### Alizarin red staining

Osteogenic differentiation and deposition of calcified matrix was determined by alizarin red staining at day 14 and day 21. For this, osteogenically differentiated and expansion medium control SMC cultivated in 96-well plates were fixated with Roti-Histofix 4% after evaluation of the metabolic activity. Prior to alizarin red staining cells were stained with 10 µg/ml Hoechst (Sigma) diluted in PBS for 10 min for quantification of the cell number. Hoechst staining per well was determined at 460 nm using an infinite M200PRO plate reader. Subsequently, wells were stained with 75 µl 0.5% alizarin red S (Sigma; in ddH_2_O, pH 4.2) for 10 min at room temperature. Wells were washed three times with ddH_2_O to remove unbound dye, further washing steps were added if needed. Plates were allowed to dry and staining was documented macroscopically using an IXU185 camera (Canon) as well as microscopically using a Leica DMIL LED with a 5× N PLAN PHO objective, equipped with a Leica DFC345 FX camera and the imaging software Leica Application Suite V3.7. Alizarin red staining of the deposited calcified matrix was quantified by absorption at 562 nm using an infinite M200PRO plate reader, following elution with cetylpyridinium chloride (Sigma; 10% solution in ddH_2_O). Blank values of cetylpyridinium chloride were subtracted and alizarin red values were normalized to the cell number determined by Hoechst staining. Values are given as mean of five replicate wells per donor and medium. To analyse the medium impact on the osteogenic differentiation of SMC, means of the quantified alizarin red values were calculated for each osteogenic differentiation medium used. Expansion medium controls of DMEM and DMEM plus supplements as well as M199 and M199 plus supplements were pooled for this quantitative analysis.

### Statistical analysis

For graphical representation, fold change calculation, and statistical analysis GraphPad Prism V8.0.0 was used. Data of each donor are expressed as mean of five replicate wells ± standard deviation. Boxplots represent the 25th, 50th, and 75th percentile and whiskers represent the range of the observations. Each point signifies a single donor. Data were analysed for normal distribution using a Shapiro-Wilk test. Due to outliers normal distribution could not be confirmed for all data sets tested, thus, for statistical comparison non-parametric testing was applied. For comparison of experimental groups during each point in time of the osteogenic differentiation in VascuLife and DMEM stained with alizarin red and for the related cell numbers determined by nuclei staining with HOECHST an unpaired Kruskal-Wallis-test with a Dunn’s post test corrected for multiple comparisons was used. For the exploratory gene expression analysis an uncorrected Dunn’s post test was applied. Level of significance were set at p < 0.05. No statistical analysis was possible for SMC osteogenically stimulated in M199 due to the limited number of experimental groups.

## Supplementary information

Supplementary information.

Supplementary movie a.

Supplementary movie b.

Supplementary movie c.

Supplementary movie d.

Supplementary movie e.

Supplementary movie f.

Supplementary movie g.

Supplementary movie h.

Supplementary movie i.

Supplementary movie j.

## Data Availability

All data generated or analysed during this study are included in this published article and its Supplementary Information Files.
