# Supplementary material for: Induced osteogenic differentiation of human smooth muscle cells as a model of vascular calcification
Source: Sci Rep. 2020 Apr 6;10:5951. doi: 10.1038/s41598-020-62568-w (PMC7136202; doi:10.1038/s41598-020-62568-w)
Supplement: Supplementary file 1 — Supplementary information. [file 41598_2020_62568_MOESM1_ESM.pdf]

Supplementary information

Induced osteogenic differentiation of human smooth muscle cells as a model of vascular calcification

Wera Pustlauk[1,2,3,4], Timm H. Westhoff[4], Luc Claeys[5], Toralf Roch[1,4,6], Sven Geißler[1,2,6*] & Nina Babel[1,4,6]

Supplementary Table S1: Cultivation media composition used for SMC isolation and expansion

Medium	Glucose [mg/l]	FCS [%]	Glutamine	Calcium [mM]	Phosphate [mmol/l] *	Additional supplements
VL	unknown	5	5 %	1.6	0.7	FGF (5 ng/ml)<br>EGF (5 ng/ml)<br>insulin (5 µg/ml)<br>ascorbic acid (50 µg/ml)
D	1000	10	1 %	1.58	1.1	-
D+S	1000	10	1 %	1.58	1.1	FGF (5 ng/ml)<br>EGF (5 ng/ml)<br>insulin (5 µg/ml)<br>ascorbic acid (50 µg/ml)
M	1000	10	0.1 g/l #	1.36	1.1	-
M+S	1000	10	0.1 g/l #	1.36	1.1	FGF (5 ng/ml)<br>EGF (5 ng/ml)<br>insulin (5 µg/ml)<br>ascorbic acid (50 µg/ml)

D - DMEM, M - M199, S - supplements (FGF, EGF, insulin, ascorbic acid), VL - VascuLife, EGF - endothelial growth factor, FGF - fibroblast growth factor, * - determined by phosphate assay, # - in medium included

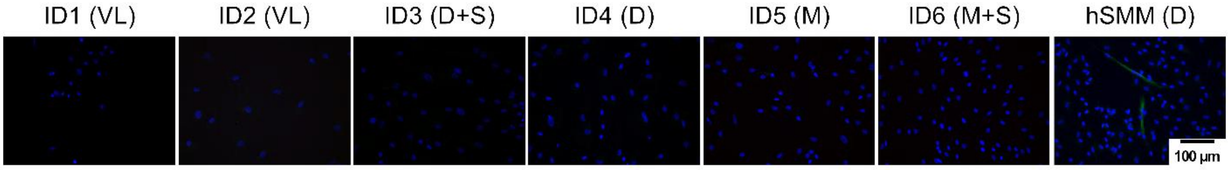

Supplementary Figure S1: Myosin heavy chain 4 expression on human SMC after expansion in different media

Cells isolated from various pathologically altered, clinical samples of different arterial origin and human skeletal muscle myoblasts (hSMM) were analysed by immunofluorescence staining for the human skeletal muscle myoblast marker myosin heavy chain 4 (green) to confirm their SMC phenotype at passage three. Myosin heavy chain 4 was labelled with the respective antibody, nuclei (blue) were visualized with DAPI. Expansion medium of each donor is given in brackets; D - DMEM, M - M199, S - supplements (FGF, EGF, insulin, ascorbic acid), VL - VascuLife.

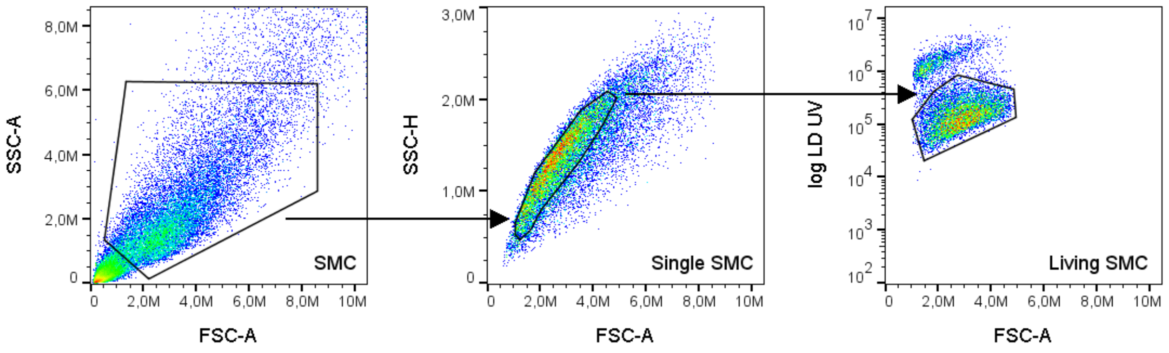

Supplementary Figure S2: Gating strategy to identify single live SMC

Gating strategy for flow cytometric surface marker analysis of cells from ID4. Dot plots are representative for all isolated cells. From left to right: Scatter plot, distinguishing cells from debris; FSC-Height (H) plotted against FSC-Area (A) to exclude cell aggregates; dead cells were labelled using the LIVE/DEAD^{TM} Fixable Blue Dead Cell Stain Kit and plotted against FSC-A to discriminate between live and dead cells. 40.000 cells of passage three to four were analysed per donor.

Supplementary Movies: Contractility of SMC varies between donors

SMC expanded in different cultivation media were functionally assessed by stimulation with 10 µM carbachol in their respective expansion medium (a-f) or in VascuLife medium after being transferred to this medium for one passage (g-j). Time lapse images of the stimulated cells were recorded for 15 min using a Leica DMI 6000B equipped with a 5x or 10x HCX PL FLUOTA objective, a Leica DFC345 FX camera and a BL-X incubator. Serial images were converted to AVI movies. Contraction assay in the respective expansion medium; (a) SMC of ID1 expanded in VL; (b) SMC of ID2 expanded in VL; (c) SMC of ID3 expanded in D; (d) SMC of ID4 expanded in D+S; (e) SMC of ID5 expanded in M; (f) SMC of ID6 expanded in M+S. Contraction assay after transfer to VascuLife medium; (g) SMC of ID3; (h) SMC of ID4; (i) SMC of ID5; (j) SMC of ID6; D - DMEM, M - M199, S - supplements (FGF, EGF, insulin, ascorbic acid), VL - VascuLife.

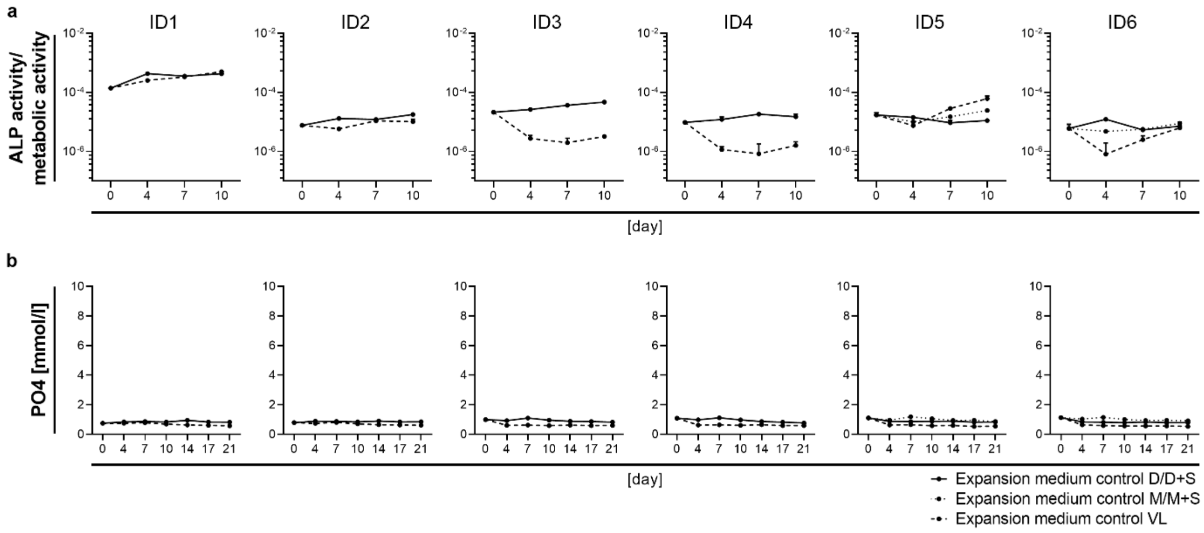

Supplementary Figure S3: Alkaline phosphatase activity and phosphate level of SMC cultured in different expansion media

(a) Alkaline phosphatase (ALP) activity of SMC cultured in VascuLife, DMEM, or M199. ALP activity was normalized to the metabolic activity of the cells determined with PrestoBlue Cell Viability Reagent. (b) Phosphate (PO4) level determined in the supernatant of SMC cultured in VascuLife, DMEM, or M199. ALP and PO4 values are given as mean of five replicate wells per donor and medium ± standard deviation; D - DMEM, M - M199, S - supplements (FGF, EGF, insulin, ascorbic acid), VL - VascuLife.

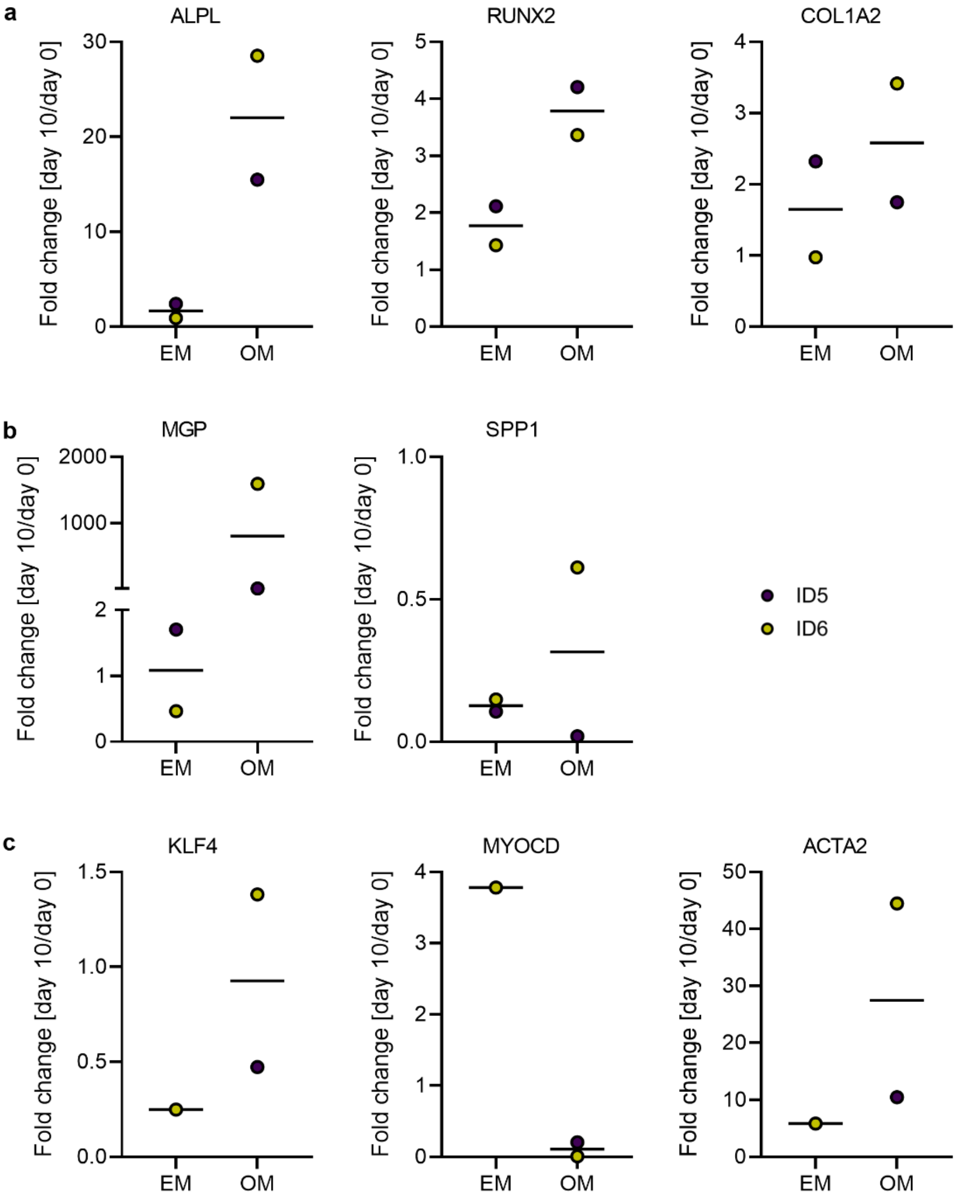

Supplementary Figure S4: Increased gene expression of osteogenic markers after stimulation in M199 substantiates an osteogenic differentiation of SMC while SMC characteristics are partially maintained

Gene expression analysis of SMC osteogenically stimulated in M199 for ten days; (a) osteogenic marker, (b) negative regulators of calcification, (c) SMC marker and transcriptional regulators. Expression was normalized to the expression of the housekeeping gene *RPL13A* and the fold change between day zero (baseline) and day ten was calculated. Expansion medium controls for M199 and M199 plus supplements were pooled for quantitative analysis. Low mRNA concentrations of ID5 in the EM control limited its analysis; M - M199, S - supplements (FGF, EGF, insulin, ascorbic acid), EM - expansion medium, OM - osteogenic medium.

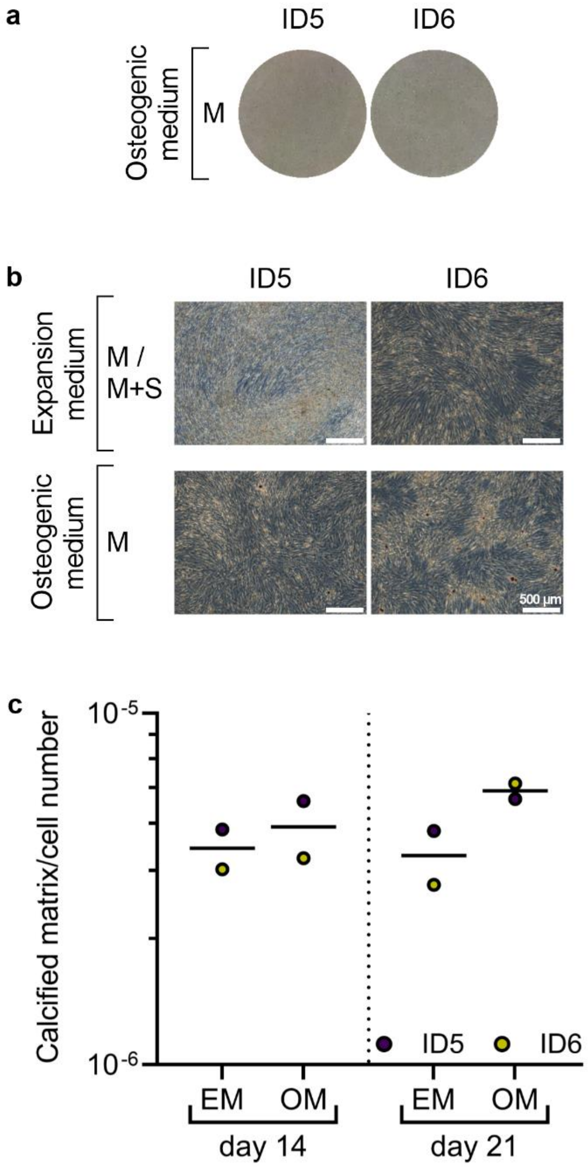

Supplementary Figure S5: SMC osteogenically stimulated in M199 deposit calcified matrix only scarcely

Alizarin red staining of calcified matrix deposited by SMC derived from ID5 and ID6 that were osteogenically stimulated in M199 for 14 and 21 days. (a) Macroscopic examination of calcified matrix deposition after 21 days of osteogenic stimulation. (b) Microscopic examination of the wells and their respective expansion media controls after 21 days of osteogenic stimulation. (c) Quantification of the deposited calcified matrix normalized to the cell number on day 14 and day 21. Expansion medium controls for M199 and M199 plus supplements were pooled for quantitative analysis. Values of the individual donors are shown as points superimposed onto the plots and are given as mean of five replicate wells per donor and medium; M - M199, S - supplements (FGF, EGF, insulin, ascorbic acid), EM - expansion medium, OM - osteogenic medium.

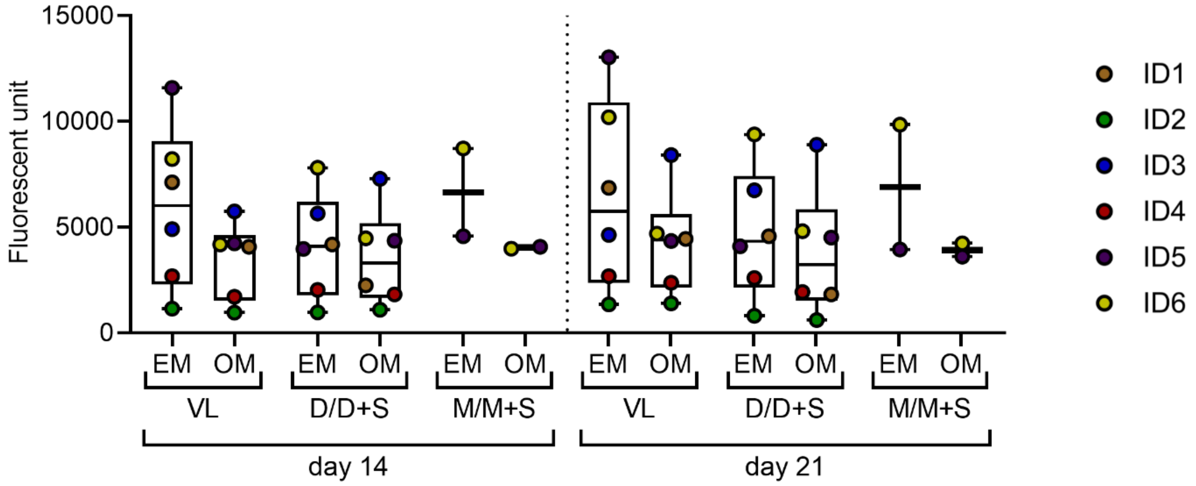

Supplementary Figure S6: SMC numbers during osteogenic differentiation

SMC were osteogenically stimulated for 14 and 21 days in VascuLife, DMEM or M199. Cell numbers were assessed by nuclei staining using HOECHST. No significant differences ($p<0.05$) between the different media, expansion medium (EM) and osteogenic medium (OM) or between day 14 and day 21 could be determined using a Kruskal-Wallis-test with an uncorrected Dunn's post test; D - DMEM, M - M199, S - supplements (FGF, EGF, insulin, ascorbic acid), VL - VascuLife.
